# Supplementary material for: TLR4 Deficiency Affects the Microbiome and Reduces Intestinal Dysfunctions and Inflammation in Chronic Alcohol-Fed Mice
Source: Int J Mol Sci. 2021 Nov 27;22(23):12830. doi: 10.3390/ijms222312830 (PMC8657603; doi:10.3390/ijms222312830)
Supplement: Supplementary file 1 [file ijms-22-12830-s001.zip › ijms-1451443-supplementary.pdf]

Supplementary Material:

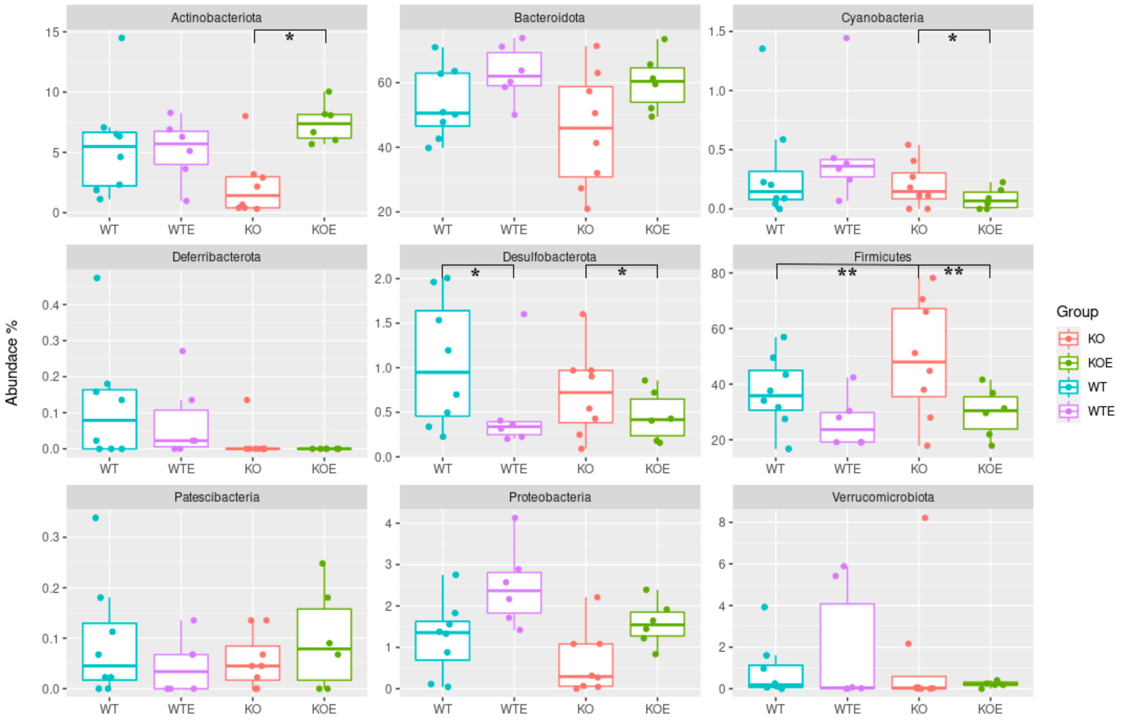

Supplementary Figure S1. Influence of alcohol and genotype in mice at the phylum level. The abundances of each phylum were assessed for significant differences between WTE vs WT, KOE vs KO and KO vs WT. Differential expression analysis was determined using *DESeq2* package and is shown in Table 2. Library normalization was performed with poscounts method and local fitting for the negative binomial distribution model. Benjamini–Hochberg method was used to perform the p-value correction. * $p < 0.05$, ** $p < 0.01$.

Supplementary Table S1. Samples used in the study with their size, total reads, final viable readings and taxonomized percentage of each taxon.

									% Taxonomized taxa				
**Group**	**Sample**	**Indexes**	**Sequence S**	**Sequence N**	**Average size**	**Total reads**	**Final reads %**	**Final reads**	**Phylum**	**Class**	**Order**	**Family**	**Genus**
Wild-Type	WT-1	S503 - N702	TATCCTCT	CGTACTAG	667	61725	64.23%	39649	100%	100%	99.75%	94.22%	47.74%
	WT-2	S503 - N701	TATCCTCT	TAAGGCGA	673	51591	68.15%	35160	100%	100%	100.00%	92.27%	53.37%
	WT-3	S503 - N703	TATCCTCT	AGGCAGAA	686	31108	62.49%	19440	100%	100%	100.00%	95.74%	50.35%
	WT-4	S503 - N704	TATCCTCT	TCCTGAGC	672	23280	58.08%	13520	100%	100%	100.00%	96.65%	53.97%
	WT-5	S503 - N706	TATCCTCT	TAGGCATG	660	24680	62.56%	15440	100%	100%	100.00%	89.45%	53.91%
	WT-6	S504 - N701	AGAGTAGA	TAAGGCGA	668	23960	62.72%	15028	100%	100%	100.00%	95.65%	52.84%
	WT-7	S504 - N702	AGAGTAGA	CGTACTAG	606	7144	62.05%	4433	100%	100%	100.00%	95.38%	50.77%
	WT-8	S504 - N701	AGAGTAGA	TAAGGCGA	630	18253	65.85%	12020	100%	100%	100.00%	93.01%	47.31%
Chronic Wild-Type	WTE-1	S503 - N705	TATCCTCT	GGACTCCT	621	20518	65.66%	13472	100%	100%	99.62%	93.23%	53.01%
	WTE-2	S503 - N703	TATCCTCT	AGGCAGAA	680	23998	56.86%	13646	100%	100%	100%	92.72%	44.66%

	WTE-3	S503 - N704	TATCCTCT	TCCTGAGC	655	21630	63.05%	13638	100%	100%	99.59%	89.30%	46.50%
	WTE-4	S503 - N706	TATCCTCT	TAGGCATG	621	18971	71.34%	13533	100%	100%	100.00%	93.30%	45.88%
	WTE-5	S502 - N704	CTCTCTAT	TCCTGAGC	625	31558	74.44%	23493	100%	100%	100.00%	91.88%	55.13%
	WTE-6	S502 - N703	CTCTCTAT	AGGCAGAA	623	21989	57.44%	12630	100%	100%	99.56%	86.40%	45.18%
TLR4-knockout	KO-1	S502 - N706	CTCTCTAT	TAGGCATG	661	89854	70.92%	63724	100%	100%	99.80%	93.08%	51.53%
	KO-2	S502 - N705	CTCTCTAT	GGACTCCT	687	99983	71.29%	71274	100%	100%	99.13%	92.36%	44.10%
	KO-3	S517 - N701	GCGTAAGA	TAAGGCGA	628	17907	60.55%	10843	100%	100%	100.00%	97.83%	48.26%
	KO-4	S517 - N702	GCGTAAGA	CGTACTAG	631	27921	63.79%	17810	100%	100%	100.00%	94.88%	51.15%
	KO-5	S517 - N703	GCGTAAGA	AGGCAGAA	647	48240	46.32%	22345	100%	100%	100.00%	96.49%	52.19%
	KO-6	S517 - N704	GCGTAAGA	TCCTGAGC	628	18126	52.18%	9459	100%	100%	99.60%	92.06%	54.76%
	KO-7	S517 - N705	GCGTAAGA	GGACTCCT	631	18686	56.23%	10508	100%	100%	100.00%	93.16%	52.63%
	KO-8	S517 - N706	GCGTAAGA	TAGGCATG	624	18654	58.61%	10933	100%	100%	100.00%	94.47%	53.46%
Chronic TLR4-knockout	KOE-1	S504 - N706	AGAGTAGA	TAGGCATG	631	16115	61.23%	9868	100%	100%	100.00%	93.96%	50.00%
	KOE-2	S504 - N703	AGAGTAGA	AGGCAGAA	617	16102	57.15%	9203	100%	100%	100.00%	97.56%	47.56%
	KOE-3	S504 - N704	AGAGTAGA	TCCTGAGC	606	13019	61.70%	8033	100%	100%	100.00%	93.96%	56.38%
	KOE-4	S504 - N705	AGAGTAGA	GGACTCCT	646	13101	62.35%	8168	100%	100%	100.00%	94.20%	45.65%
	KOE-5	S502 - N701	CTCTCTAT	TAAGGCGA	658	16241	70.75%	11491	100%	100%	100.00%	95.60%	49.06%
	KOE-6	S502 - N702	CTCTCTAT	CGTACTAG	659	48185	61.10%	29443	100%	100%	100.00%	93.79%	50.31%

Supplementary Table S2. Statistical analysis of Gram positive/negative ratios. A Kruskal-Wallis test with a Dunn's multiple comparison test was performed, p-values were corrected by Sidak method.

Comparison	Z-score	Raw p-value	Adjusted p-value
KO - KOE	-1.359959	0.08692137	0.42050584
KO - WT	-0.7597883	0.22369056	0.78111842
WT - WTE	-15.944353	0.05541929	0.28971207

Supplementary Table S3. Differential expression in bacteria taxa among WTE vs WT, KOE vs KO and KO vs WT. Tests for differential expression were made using *DESeq2* package. Only ASVs with an adjusted P-value < 0.01 are shown.

	baseMean	log2FoldChange	lfcSE	stat	pvalue	padj	Kingdom	Phylum	Class	Order	Family	Genus
**WTE vs WT**												
ASV19	109.944	22.8434	1.975498	11.56338	6.32E-31	3.39E-28	Bacteria	Bacteroidota	Bacteroidia	Bacteroidales	Prevotellaceae	Alloprevotella
ASV76	41.942	22.8085	2.312726	9.862166	6.07E-23	2.17E-20	Bacteria	Bacteroidota	Bacteroidia	Bacteroidales	Muribaculaceae	NA
ASV16	132.169	22.7102	1.617544	14.03992	8.88E-45	9.53E-42	Bacteria	Bacteroidota	Bacteroidia	Bacteroidales	Prevotellaceae	Alloprevotella
ASV139	21.893	22.4117	3.051977	7.343348	2.08E-13	5.59E-11	Bacteria	Bacteroidota	Bacteroidia	Bacteroidales	Muribaculaceae	NA
ASV61	53.822	8.5337	1.875343	4.550462	5.35E-06	0.000638	Bacteria	Firmicutes	Clostridia	Lachnospirales	Lachnospiraceae	Lachnospiraceae_NK4A136_group
ASV94	39.708	-20.5259	4.209332	-4.87628	1.08E-06	0.000145	Bacteria	Firmicutes	Clostridia	Lachnospirales	Lachnospiraceae	Lachnospiraceae_NK4A136_group
ASV116	33.492	-20.6666	3.858716	-5.35582	8.52E-08	1.52E-05	Bacteria	Firmicutes	Clostridia	Lachnospirales	Lachnospiraceae	Lachnospiraceae_NK4A136_group
ASV390	6.245	-20.8273	4.209199	-4.94805	7.50E-07	0.000115	Bacteria	Firmicutes	Clostridia	Lachnospirales	Lachnospiraceae	Lachnoclostridium
ASV120	28.251	-22.5720	4.206935	-5.36542	8.08E-08	1.52E-05	Bacteria	Firmicutes	Clostridia	Lachnospirales	Lachnospiraceae	Lachnoclostridium
**KOE vs KO**												
ASV185	21.449	20.6458	3.137021	6.581352	4.66E-11	4.09E-09	Bacteria	Firmicutes	Bacilli	Erysipelotrichales	Erysipelotrichaceae	Dubosiella
ASV199	20.046	20.5954	3.098727	6.646412	3.00E-11	3.30E-09	Bacteria	Firmicutes	Bacilli	Erysipelotrichales	Erysipelotrichaceae	Dubosiella

ASV46	94.293	8.7036	1.246333	6.983398	2.88E-12	4.22E-10	Bacteria	Firmicutes	Bacilli	Erysipelotrichales	Erysipelotrichaceae	Faecalibaculum
ASV67	79.088	7.0365	1.377357	5.10869	3.24E-07	1.29E-05	Bacteria	Firmicutes	Bacilli	Erysipelotrichales	Erysipelotrichaceae	Faecalibaculum
ASV99	31.531	-7.1986	1.337211	-5.38328	7.31E-08	4.01E-06	Bacteria	Bacteroidota	Bacteroidia	Bacteroidales	Rikenellaceae	Rikenellaceae_RC9_gut_group
ASV113	36.466	-7.4630	1.641944	-4.54522	5.49E-06	0.000172	Bacteria	Firmicutes	Clostridia	Lachnospirales	Lachnospiraceae	NA
ASV112	31.103	-8.0501	1.789651	-4.49812	6.86E-06	0.000201	Bacteria	Firmicutes	Clostridia	Lachnospirales	Lachnospiraceae	Lachnospiraceae_UCG-006
ASV123	31.138	-8.4687	2.002098	-4.2299	2.34E-05	0.000641	Bacteria	Firmicutes	Clostridia	Lachnospirales	Lachnospiraceae	Lachnoclostridium
ASV96	36.447	-8.4963	2.026033	-4.19355	2.75E-05	0.000709	Bacteria	Firmicutes	Clostridia	Lachnospirales	Lachnospiraceae	Lachnospiraceae_UCG-006
ASV119	33.140	-8.6374	2.274369	-3.79773	0.000146	0.003562	Bacteria	Firmicutes	Clostridia	Lachnospirales	Lachnospiraceae	Lachnoclostridium
ASV313	4.721	-19.9258	4.207599	-4.73566	2.18E-06	7.37E-05	Bacteria	Firmicutes	Clostridia	Lachnospirales	Lachnospiraceae	Lachnospiraceae_NK4A136_group
ASV284	10.145	-20.4061	4.208953	-4.84827	1.25E-06	4.56E-05	Bacteria	Firmicutes	Bacilli	Erysipelotrichales	Erysipelatoclostridiaceae	Erysipelatoclostridium
ASV95	39.620	-21.2693	2.43665	-8.72893	2.57E-18	1.13E-15	Bacteria	Firmicutes	Clostridia	Lachnospirales	Lachnospiraceae	NA
ASV186	18.830	-21.4070	3.325325	-6.43757	1.21E-10	8.88E-09	Bacteria	Firmicutes	Clostridia	Lachnospirales	Lachnospiraceae	NA
ASV260	10.512	-21.4602	3.586402	-5.98376	2.18E-09	1.37E-07	Bacteria	Firmicutes	Clostridia	Oscillospirales	Ruminococcaceae	NA
ASV371	10.858	-21.5853	4.207246	-5.13051	2.89E-07	1.27E-05	Bacteria	Firmicutes	Clostridia	Lachnospirales	Lachnospiraceae	Lachnospiraceae_NK4A136_group
ASV138	25.060	-21.5973	2.72174	-7.93512	2.10E-15	4.62E-13	Bacteria	Firmicutes	Clostridia	Lachnospirales	Lachnospiraceae	NA
ASV151	18.286	-22.3296	4.206732	-5.30807	1.11E-07	5.40E-06	Bacteria	Firmicutes	Clostridia	Lachnospirales	Lachnospiraceae	Roseburia
**KO vs WT**												
ASV16	132.169	25.7608	1.51867	16.96275	1.55E-64	5.72E-62	Bacteria	Bacteroidota	Bacteroidia	Bacteroidales	Prevotellaceae	Alloprevotella
ASV19	109.944	25.5490	1.846483	13.83656	1.53E-43	2.83E-41	Bacteria	Bacteroidota	Bacteroidia	Bacteroidales	Prevotellaceae	Alloprevotella
ASV76	41.942	23.9317	2.156489	11.09754	1.29E-28	1.59E-26	Bacteria	Bacteroidota	Bacteroidia	Bacteroidales	Muribaculaceae	NA
ASV139	21.893	23.5059	2.836938	8.285647	1.17E-16	1.08E-14	Bacteria	Bacteroidota	Bacteroidia	Bacteroidales	Muribaculaceae	NA
ASV248	13.658	21.4170	3.887979	5.508527	3.62E-08	9.54E-07	Bacteria	Firmicutes	Clostridia	Lachnospirales	Lachnospiraceae	Lachnospiraceae_NK4A136_group
ASV50	52.731	9.5427	2.384325	4.002269	6.27E-05	0.001052	Bacteria	Firmicutes	Clostridia	Lachnospirales	Lachnospiraceae	Lachnospiraceae_NK4A136_group
ASV61	53.822	9.2664	1.756595	5.275181	1.33E-07	3.26E-06	Bacteria	Firmicutes	Clostridia	Lachnospirales	Lachnospiraceae	Lachnospiraceae_NK4A136_group
ASV142	22.881	8.1593	2.028308	4.022696	5.75E-05	0.001011	Bacteria	Firmicutes	Clostridia	Oscillospirales	Ruminococcaceae	NA
ASV195	24.671	7.5670	1.793977	4.218028	2.46E-05	0.000479	Bacteria	Firmicutes	Clostridia	Lachnospirales	Lachnospiraceae	Lachnospiraceae_NK4A136_group
ASV217	16.149	7.3888	1.8037	4.096482	4.19E-05	0.000774	Bacteria	Firmicutes	Clostridia	Oscillospirales	Oscillospiraceae	Papillibacter
ASV181	19.390	7.2692	1.963839	3.701507	0.000214	0.003438	Bacteria	Firmicutes	Clostridia	Lachnospirales	Lachnospiraceae	NA
ASV3	423.935	-1.8668	0.5249	-3.55651	0.000376	0.005778	Bacteria	Bacteroidota	Bacteroidia	Bacteroidales	Muribaculaceae	NA
ASV4	364.653	-1.9266	0.545027	-3.53484	0.000408	0.006023	Bacteria	Bacteroidota	Bacteroidia	Bacteroidales	Muribaculaceae	NA
ASV67	79.088	-5.5732	1.285676	-4.33483	1.46E-05	0.000299	Bacteria	Firmicutes	Bacilli	Erysipelotrichales	Erysipelotrichaceae	Faecalibaculum
ASV46	94.293	-7.4539	1.179956	-6.31708	2.67E-10	8.94E-09	Bacteria	Firmicutes	Bacilli	Erysipelotrichales	Erysipelotrichaceae	Faecalibaculum
ASV152	31.769	-8.9641	1.527846	-5.86714	4.43E-09	1.26E-07	Bacteria	Bacteroidota	Bacteroidia	Bacteroidales	Muribaculaceae	NA
ASV164	31.004	-9.0038	1.378957	-6.52945	6.60E-11	2.71E-09	Bacteria	Bacteroidota	Bacteroidia	Bacteroidales	Muribaculaceae	NA
ASV70	59.912	-9.6623	1.639738	-5.89258	3.80E-09	1.17E-07	Bacteria	Bacteroidota	Bacteroidia	Bacteroidales	Prevotellaceae	Alloprevotella
ASV48	76.783	-9.9230	1.91765	-5.17455	2.28E-07	4.96E-06	Bacteria	Bacteroidota	Bacteroidia	Bacteroidales	Muribaculaceae	NA
ASV57	70.728	-9.9494	1.474107	-6.74943	1.48E-11	7.82E-10	Bacteria	Bacteroidota	Bacteroidia	Bacteroidales	Prevotellaceae	Alloprevotella
ASV33	92.148	-10.1804	1.946326	-5.23055	1.69E-07	3.90E-06	Bacteria	Bacteroidota	Bacteroidia	Bacteroidales	Muribaculaceae	NA
ASV199	20.046	-20.9697	2.879616	-7.28213	3.29E-13	2.02E-11	Bacteria	Firmicutes	Bacilli	Erysipelotrichales	Erysipelotrichaceae	Dubosiella
ASV299	11.891	-21.1414	3.247343	-6.51038	7.50E-11	2.77E-09	Bacteria	Bacteroidota	Bacteroidia	Bacteroidales	Rikenellaceae	Alistipes
ASV185	21.449	-21.3448	2.914615	-7.32337	2.42E-13	1.78E-11	Bacteria	Firmicutes	Bacilli	Erysipelotrichales	Erysipelotrichaceae	Dubosiella
ASV263	16.112	-21.8953	3.341976	-6.55161	5.69E-11	2.63E-09	Bacteria	Actinobacteriota	Actinobacteria	Bifidobacteriales	Bifidobacteriaceae	Bifidobacterium
